# Reply: Assessment of therapeutic response in patients with metastatic skeletal disease: suggested modifications for the MDA response classification criteria

**DOI:** 10.1038/sj.bjc.6605826

**Published:** 2010-07-27

**Authors:** T Hamaoka, C M Costelloe, J E Madewell, P Liu, G N Hortobagyi, N T Ueno

**Affiliations:** 1 Department of Stem Cell Transplantation and Cellular Therapy, The University of Texas MD Anderson Cancer Center, Houston, TX, USA; 2 Department of Breast Surgical Oncology, St Luke's International Hospital, Tokyo, Japan; 3 Department of Diagnostic Radiology, The University of Texas MD Anderson Cancer Center, Houston, TX, USA; 4 Department of Biostatistics and Applied Mathematics, The University of Texas MD Anderson Cancer Center, Houston, TX, USA; 5 Department of Breast Medical Oncology, The University of Texas MD Anderson Cancer Center, Houston, TX, USA

**Sir,**

We appreciate the constructive comments of Dr Vassiliou and Dr Andreopoulos to our publication ‘Tumour response interpretation with new tumour response criteria *vs* the World Health Organisation criteria in patients with bone-only metastatic breast cancer’ (Hamaoka *et al*, 2010). We agree that the MDA criteria might become more accurate if they encompassed a greater scope of quantitative measurements. However, we intentionally avoided discussing developing criteria based exclusively on quantitative measurements because the quantitative measurements of radiographic changes in bone have historically proven to be extremely difficult, would therefore obligate us to exclude a large number of bone metastases as ‘unmeasurable’ and thus limit the use of our criteria.

We have conducted a prospective clinical trial of bone tumour imaging comparing the MDA criteria with the International Union Against Cancer and World Health Organization criteria. The trial results are now being analysed, and we will soon prepare a report on our findings. From the preliminary results of the prospective data, we once again recognise the importance of change in lesion density (with computed tomography) and/or signal intensity (with magnetic resonance imaging, MRI) as an indicator of response to therapy.

In addition to measuring the sum of the largest perpendicular dimensions of measurable bone lesions, another potential way to incorporate quantitative measurements into the MDA response criteria would be to include response measurements within a region of interest of specifically defined size to determine Houndsfield unit change on CT. However, the precision of repeated measurements is a major concern regarding the use of quantitative CT in our response criteria. It has been shown that many variables such as scanner type (e.g., 4 as compared with 16 channels), pitch, gantry rotation speed, table speed, table height, and X-ray tube temperature can affect the precision of bone mineral density measurements, and variability of 1.4–3.6% has been reported (Bligh *et al*, 2009). Small differences in precision can be cumulative and lead to erroneous results when allowed to accumulate over several time points, unless painstaking quality control methods are used, such as scanning each patient using the same type of scanner (preferably the same exact scanner) with the same imaging parameters. This can be logistically difficult in a large practice. Similar issues arise regarding the precision of repeated MRI measurements, and scan parameters must also be uniform to permit adequate comparison of quantitative MRI results using standardised MR scan parameters over many time points on different scans. Another possibility is to measure changes in the standardised uptake value on fluorodeoxyglucose positron emission tomography–computed tomography. Regardless of the methods of quantification, any changes to the MDA response criteria would need to be tested in prospective clinical trials by comparison with the current MDA criteria to determine whether adding additional quantitative indicators would be truly useful in defining a clinically relevant response. It is the opinion of the authors that the qualitative portions of the MDA criteria constitute their greatest strength by allowing outcome determination of a wide variety of bone metastases in a simple and straightforward manner.
